# Supplementary figures and images for: Implementation research protocol on the national community health policy in Guinea: A sequential mixed-methods study using a decision space approach
Source: PLoS One. 2023 Jan 20;18(1):e0280651. doi: 10.1371/journal.pone.0280651 (PMC9858093; doi:10.1371/journal.pone.0280651)

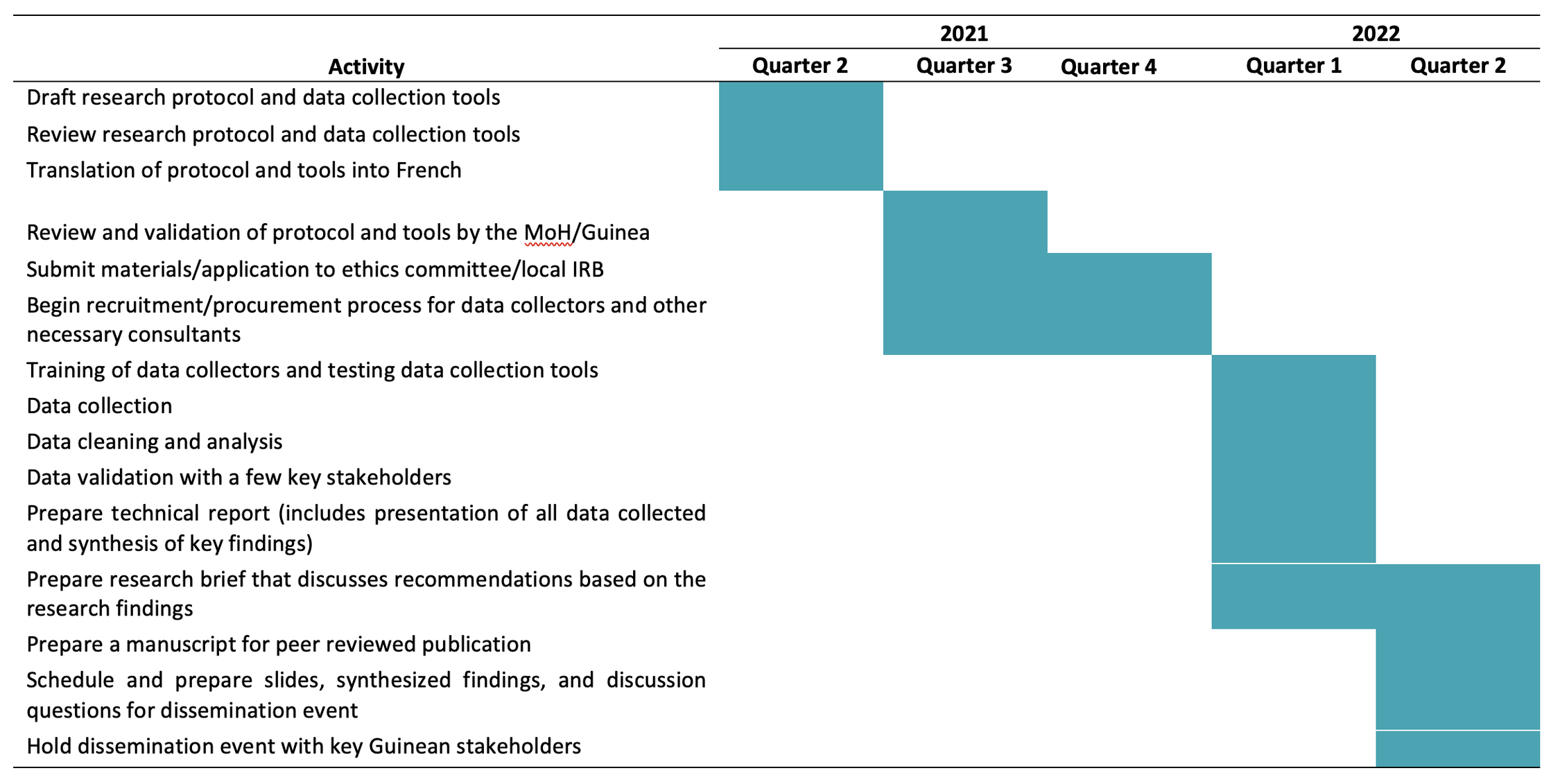

Supplement: S1 Fig — (TIF) [file pone.0280651.s001.tif]
