# Supplementary material for: Implementation research protocol on the national community health policy in Guinea: A sequential mixed-methods study using a decision space approach
Source: PLoS One. 2023 Jan 20;18(1):e0280651. doi: 10.1371/journal.pone.0280651 (PMC9858093; doi:10.1371/journal.pone.0280651)
Supplement: S1 Table — National level stakeholders are only minimally targeted by the survey because they fit more with the qualitative component where in-depth interviews are more suitable to capture a wealth of information on the policy design and rollout. (DOCX) [file pone.0280651.s002.docx]

***S1 Table****

| **Target group** | **Estimated sample** |
| --- | --- |
| **National level**   - Ministerial departments (Territorial Administration & Decentralization; Finances; Budget; Agriculture, Vocational schools; Social Action; and Mining) - MFPREMA (Ministère de Fonction Publique et de la Réforme de l’Administration), MATD, MoH technical and financial partners - NGOs - Parliamentarians | N=15-20 |
| **Regional and district levels**   - Regional health team (Director, Community health specialist, Disease control specialist, Planning & research officer) - Governor - District health team - Prefectural Officer (Prefet) - Mayor - NGOs regional and district offices - Regional and district offices of Ministry of social affairs, ANAFIC - Religious leaders - Civil society leaders | N=10-12 at regional level  N=10-12 at district level |
| **Local level (rural commune)**   - Head of health center - Mayor of commune - Health and hygiene committee - Village health committees - Religious leaders - Community-based organizations - ASC/RECO - Traditional medicine practitioners | N=20 per commune |

*National level stakeholders are only minimally targeted by the survey because they fit more with the qualitative component where in-depth interviews are more suitable to capture a wealth of information on the policy design and rollout.
