# Supplementary material for: Implementation research protocol on the national community health policy in Guinea: A sequential mixed-methods study using a decision space approach
Source: PLoS One. 2023 Jan 20;18(1):e0280651. doi: 10.1371/journal.pone.0280651 (PMC9858093; doi:10.1371/journal.pone.0280651)
Supplement: S2 Table — Regions selected based on demographic and health indicators. (DOCX) [file pone.0280651.s003.docx]

***S2 Table***

| **Characteristics** | **Boké** | **Faranah** | **Kankan** | **Kindia*** | **Labé*** | **Mamou*** | **N'Zérékoré*** |
| --- | --- | --- | --- | --- | --- | --- | --- |
| Total population size | 1,330,078 | 1,156,312 | 2,409,866 | 1,916,277 | 1,219,391 | 897,517 | 1,938,227 |
| Total rural population | 975,735 | 890,147 | 1,902,296 | 1,219,840 | 1,074,609 | 767,854 | 1,460,243 |
| Proportion of rural population | 73% | 77% | 79% | 64% | 88% | 86% | 75% |
| Proportion of Population under the national poverty line | 12% | 22% | 6% | 18% | 23% | 12% | 12% |
| Adult literacy rate | 41% | 23% | 29% | 37% | 25% | 40% | 31% |
| Effective immunization coverage of children 12-23 months | 17% | 20% | 36% | 12% | 8% | 13% | 35% |
| Anemia in children 6-59 months | 69% | 78% | 77% | 74% | 71% | 76% | 76% |
| Assisted birth delivery | 46% | 38% | 56% | 51% | 34% | 42% | 64% |

*Regions selected based on demographic and health indicators
