# Supplementary material for: Implementation research protocol on the national community health policy in Guinea: A sequential mixed-methods study using a decision space approach
Source: PLoS One. 2023 Jan 20;18(1):e0280651. doi: 10.1371/journal.pone.0280651 (PMC9858093; doi:10.1371/journal.pone.0280651)
Supplement: S3 Table — (DOCX) [file pone.0280651.s004.docx]

***S3 Table***

|  | **Intervention** | | **Control** |  |
| --- | --- | --- | --- | --- |
| **Selected study Region** | **Convergence Communes** | **Communes with functioning PNSC** | **Communes with no functioning PNSC** | **Total** |
| **Kindia^1^** |  |  |  |  |
| *Current situation* | 4 | 26 | 15 | 45 |
| *Study sample* | 1 | 4 | 2 | 7 |
| **Mamou** |  |  |  |  |
| *Current situation* | 2 | 31 | 3 | 36 |
| *Study sample* | 1 | 5 | 1 | 7 |
| **Labé** |  |  |  |  |
| *Current situation* | 10 | 0 | 43 | 53 |
| *Study sample* | 2 | 0 | 4 | 6 |
| **N'Zérékoré** |  |  |  |  |
| *Current situation* | 9 | 0 | 57 | 66 |
| *Study sample* | 2 | 0 | 5 | 7 |

^1^ *In Kindia, we plan to select four communes with functioning community health policy to compensate for their absence in the other three targeted regions and to ensure their representativity in the sample. One control commune will complete the sample to six rural communes.*
