# Supplementary material for: Implementation research protocol on the national community health policy in Guinea: A sequential mixed-methods study using a decision space approach
Source: PLoS One. 2023 Jan 20;18(1):e0280651. doi: 10.1371/journal.pone.0280651 (PMC9858093; doi:10.1371/journal.pone.0280651)
Supplement: S4 Table — (DOCX) [file pone.0280651.s005.docx]

***S4 Table***

| **Variable type** | **Variables** | **Data sources** |
| --- | --- | --- |
| **Independent variable of interest** | Type of commune | DNSCMT |
| **Covariates** | Population, urbanization, income, health workers ratio, disease burden and health indicators | Demographic and Health Survey and INSS |
| **Dependent variable** | Degree of decision space; capacity; and level of accountability | Quantitative decision space survey |
