# Supplementary material for: Implementation research protocol on the national community health policy in Guinea: A sequential mixed-methods study using a decision space approach
Source: PLoS One. 2023 Jan 20;18(1):e0280651. doi: 10.1371/journal.pone.0280651 (PMC9858093; doi:10.1371/journal.pone.0280651)
Supplement: S6 Table — (DOCX) [file pone.0280651.s007.docx]

***S6 Table***

| **Target group** | **Data collection technique** | **Total maximum sample** |
| --- | --- | --- |
| **National level**   - MoH: Director, Deputy directors, Head of services involved in community health policy - MATD: divisions and services involved in community policy - Ministry of Social Affairs: divisions and services involved in community policy - Development partners: UNICEF, CRS, Jhpiego, World Bank/GFF, USAID, GIZ, Global Fund, EU - Religious leaders - Civil society leaders | FGDs | 20-25 FGDs across all levels |
| **Regional level**   - Regional health team - Governor - Religious leaders - Civil society leaders - NGOs regional offices - Regional offices of Ministry of Social Affairs, ANAFIC | FGDs |  |
| **District level**   - District health team - Prefectural Officer (Préfet) - Mayor (urban communes) - NGOs district offices - District offices of Ministry of Social Affairs, ANAFIC - Civil society leaders - Religious leaders | FGD |  |
| **Local level (Rural Commune)**   - Head of health center - Mayor of rural commune - Health and Hygiene committee - ASC/RECO - Health providers - Community members | FGD  IDI | 25 IDIs |
