# Supplementary material for: Implementation research protocol on the national community health policy in Guinea: A sequential mixed-methods study using a decision space approach
Source: PLoS One. 2023 Jan 20;18(1):e0280651. doi: 10.1371/journal.pone.0280651 (PMC9858093; doi:10.1371/journal.pone.0280651)
Supplement: Attachment — Submitted filename: Rebuttal letter_PLOS ONE_ HSSA IR in Guinea_rev.docx [file pone.0280651.s008.docx]

**Chulaporn Limwattananon, Ph.D.**

Academic Editor

PLOS ONE

Manuscript ref. num: PONE-D-22-10462

December 07, 2022

**Dear Editor,**

We are very grateful to the Editor and the reviewer for the comments they provided on our paper entitled *“Implementation research protocol on the National Community Health Policy in Guinea: A sequential mixed-methods study using a decision space approach”*. We have received two sets of comments from the Editor : (i) August 4^th^, 2022, and (ii) October 10^th^, 2022. We have addressed all those comments and organized our responses by date of reception of those comments. We feel that these revisions have significantly improved our protocol content. Therefore, please find our point-by-point response below to each comment from the Academic editor and the reviewers in bold font followed by our responses in italic. We have also updated the manuscript as requested*.*

***Comments received on August 4^th^, 2022 (page 1 to 5)***

**RESPONSE TO THE ACADEMIC EDITOR COMMENTS :**

**1. Please ensure that your manuscript meets PLOS ONE's style requirements, including those for file naming. The PLOS ONE style templates can be found at**

[**https://journals.plos.org/plosone/s/file?id=wjVg/PLOSOne_formatting_sample_main_body.pdf**](https://journals.plos.org/plosone/s/file?id=wjVg/PLOSOne_formatting_sample_main_body.pdf) **and**

[**https://journals.plos.org/plosone/s/file?id=ba62/PLOSOne_formatting_sample_title_authors_affiliations.pdf**](https://journals.plos.org/plosone/s/file?id=ba62/PLOSOne_formatting_sample_title_authors_affiliations.pdf)**.**

*We have formatted the protocol accordingly.*

**2. We note that the grant information you provided in the ‘Funding Information’ and ‘Financial Disclosure’ sections do not match.**

**When you resubmit, please ensure that you provide the correct grant numbers for the awards you received for your study in the ‘Funding Information’ section.**

*Funding for this study was provided by the United States Agency for International Development (USAID). Funding was provided by USAID to the organization Results for Development (R4D) under the Health Systems Strengthening Accelerator Project under the grant number No. 7200-AA-18CA-00037. The grant information can be found at the following address* [*https://govtribe.com/award/federal-grant-award/cooperative-agreement-7200aa18ca00037*](https://govtribe.com/award/federal-grant-award/cooperative-agreement-7200aa18ca00037)*. None of the author received a grant received a grand with his/her name. We have addressed it during the submission. This comment has also been addressed in the additional comments received later (October 10). Please see below (page 6, comment 1).*

**3.Your ethics statement should only appear in the Methods section of your manuscript. If your ethics statement is written in any section besides the Methods, please delete it from any other section.**

*We confirm that this is already part of the methods section (see lines 492 -504) of the clean copy of the revised version*

**4. Please review your reference list to ensure that it is complete and correct. If you have cited papers that have been retracted, please include the rationale for doing so in the manuscript text, or remove these references and replace them with relevant current references. Any changes to the reference list should be mentioned in the rebuttal letter that accompanies your revised manuscript. If you need to cite a retracted article, indicate the article’s retracted status in the References list and also include a citation and full reference for the retraction notice**.

*We have revised the list of references. We originally had 48 and now we just have 47. This is due to an error when referencing the first version submitted to your journal. We have now found that the original 47 reference was not retained in the protocol.*

**Note: HTML markup is below. Please do not edit.**

*We did not understand this note*

**RESPONSE TO THE REVIEWERS’ COMMENTS**

**1. Does the manuscript provide a valid rationale for the proposed study, with clearly identified and justified research questions?**

**The research question outlined is expected to address a valid academic problem or topic and contribute to the base of knowledge in the field.**

**Reviewer #1: Yes**

**Reviewer #2: Yes**

*We thank the reviewer for this appreciation*

**2. Is the protocol technically sound and planned in a manner that will lead to a meaningful outcome and allow testing the stated hypotheses?

The manuscript should describe the methods in sufficient detail to prevent undisclosed flexibility in the experimental procedure or analysis pipeline, including sufficient outcome-neutral conditions (e.g. necessary controls, absence of floor or ceiling effects) to test the proposed hypotheses and a statistical power analysis where applicable. As there may be aspects of the methodology and analysis which can only be refined once the work is undertaken, authors should outline potential assumptions and explicitly describe what aspects of the proposed analyses, if any, are exploratory.**

**Reviewer #1: Yes**

**Reviewer #2: Yes**

*Thank you for this comment*

**3. Is the methodology feasible and described in sufficient detail to allow the work to be replicable?


Descriptions of methods and materials in the protocol should be reported in sufficient detail for another researcher to reproduce all experiments and analyses. The protocol should describe the appropriate controls, sample size calculations, and replication needed to ensure that the data are robust and reproducible.**

**Reviewer #1: Yes**

**Reviewer #2: Yes**

*Thank you for this comment*

**4. Have the authors described where all data underlying the findings will be made available when the study is complete?**
**The** [PLOS Data policy](https://journals.plos.org/plosone/s/materials-and-software-sharing) **requires authors to make all data underlying the findings described in their manuscript fully available without restriction, with rare exception, at the time of publication. The data should be provided as part of the manuscript or its supporting information, or deposited to a public repository. For example, in addition to summary statistics, the data points behind means, medians and variance measures should be available. If there are restrictions on publicly sharing data—e.g. participant privacy or use of data from a third party—those must be specified.**

**Reviewer #1: No**

**Reviewer #2: Yes**

*We agree with the reviewer that the information we provided at this stage was not clear. In fact, no datasets were generated or analyzed during the study design time. All relevant data from this study will be made available upon study completion.*

**5. Is the manuscript presented in an intelligible fashion and written in standard English?

PLOS ONE does not copyedit accepted manuscripts, so the language in submitted articles must be clear, correct, and unambiguous. Any typographical or grammatical errors should be corrected at revision, so please note any specific errors here.**

**Reviewer #1: Yes**

**Reviewer #2: Yes**

*Thank you for this comment*

**6. Review Comments to the Author**

**Reviewer #1: This is a well written protocol. However, I have some concerns about the role of the Government and the funder in the research. As I understand your proposal the research is being funded by USAID and the research is for the Government to inform revisions to the National Community Health Policy to improve its rollout and effectiveness. Two of the authors are employed by the Ministry of Health in Guinea. You need to reflect more carefully how independent the research will be. Do the Government employees have any conflict of interest? Will the research be independent of influence by the funder and/or the Government?
More minor points:**

*Thank for this remark. This research led by researchers from the University of Conakry who have proven their scientific integrity in conducting similar evaluations in Guinea. We have already confirmed in the cover letter and submitting process that none of the authors has declared competing or conflict of interest. We confirm that this research will be independent of influence by the funder and/or the Government.*

**1.you say the RECO are volunteers and then say they have a salary of 450,000GNF per month - are they volunteers or employees?**

*We thank the reviewer for his comments. Yes, we confirm that RECOs are community volunteers who devote some, but not all, of their time to community health activities, unlike CHWs who are recruited and dedicate their time to community health work. RECOs do not receive a salary but monthly incentives.*

**2.In the qualitative research you say that two IDIs will be interviewed but I think that you list three roles?**

*We thank the reviewer for this remark. We have addressed it (see line 483).*

**Reviewer #2: Thank you to the authors for a well written manuscript. This will provide valuable data in decentralisation of community health worker programmes and perhaps ways in which the programme may be strengthened and made sustainable.**

*We thank the reviewer for his/her kind comments*

**7. PLOS authors have the option to publish the peer review history of their article (**[**what does this mean?**](https://journals.plos.org/plosone/s/editorial-and-peer-review-process#loc-peer-review-history)**). If published, this will include your full peer review and any attached files.**

**If you choose “no”, your identity will remain anonymous, but your review may still be made public.**

**Do you want your identity to be public for this peer review? For information about this choice, including consent withdrawal, please see our Privacy Policy.**

**Reviewer #1: No**

**Reviewer #2: No**

*We respect the reviewers’ choice not to have their identity published*

***Comments received on October 10^th^, 2022 (page 6)***

**1. We note that the grant information you provided in the ‘Funding Information’ and ‘Financial Disclosure’ sections do not match.**

*This study was supported by the USAID through a grant (No. 7200-AA-18CA-00037). We have provided the right grand number during the submission.*

**2. Thank you for stating the following financial disclosure:**

"[https://www.usaid.gov/
The funder reviewed the protocol before it submission](https://www.usaid.gov/%0bThe%20funder%20reviewed%20the%20protocol%20before%20it%20submission)"

**At this time, please address the following queries:**

**a) Please clarify the sources of funding (financial or material support) for your study. List the grants or organizations that supported your study, including funding received from your institution.**

*Funding for this study was provided by the United States Agency for International Development (USAID). Funding was provided by USAID to the organization Results for Development (R4D) under the Health Systems Strengthening Accelerator Project.* 

**b) State what role the funders took in the study. If the funders had no role in your study, please state: “The funders had no role in study design, data collection and analysis, decision to publish, or preparation of themanuscript.”**

*USAID was involved in reviewing, validating, and approving the study design. They reviewed and provided comments on the protocol/manuscript before submission. They also review findings and were consulted for approval in the decision to publish.*

**c) If any authors received a salary from any of your funders, please state which authors and which funders.**

*See below*.

**d) If you did not receive any funding for this study, please state: “The authors received no specific funding for this work.”**

*The authors received no specific funding for this work. USAID provided funding to Results for Development (R4D), and employees and consultants of R4D carried out this study.*
