# Supplementary material for: Implementation research protocol on the national community health policy in Guinea: A sequential mixed-methods study using a decision space approach
Source: PLoS One. 2023 Jan 20;18(1):e0280651. doi: 10.1371/journal.pone.0280651 (PMC9858093; doi:10.1371/journal.pone.0280651)
Supplement: Attachment — Submitted filename: Rebuttal letter_PLOS ONE_ HSSA IR in Guinea_rev.docx [file pone.0280651.s009.docx]

**Chulaporn Limwattananon, Ph.D.**

Academic Editor

PLOS ONE

Manuscript ref. num: PONE-D-22-10462

December 30, 2022

**Dear Editor,**

We are very grateful to the Editor for considering publishing our protocol entitled *“Implementation research protocol on the National Community Health Policy in Guinea: A sequential mixed-methods study using a decision space approach”* in your esteemed Journal. Thank you for the different comments to which we have provided responses. We feel that these revisions have significantly improved our protocol content. Therefore, please find our point-by-point response below to each comment in bold font followed by our responses in italic. We have also updated the manuscript as requested*.*

***Comments received on August 4^th^, 2022 (page 1 to 5)***

RESPONSE TO THE ACADEMIC EDITOR’S COMMENTS **:**

**Thank you for submitting your manuscript to PLOS ONE. After careful consideration, we feel that it has merit but does not fully meet PLOS ONE’s publication criteria as it currently stands. Therefore, we invite you to submit a revised version of the manuscript that addresses the points raised during the review process.**

*We thank the Editor for this positive remark. We have improved the protocol content.*

**Exhaustive lists of abbreviations (e.g., ANC, ASCs, RECOs, SERACCO, MATD, MoH, MOH, PNSC, DNSCMT, IEC/SBC, RDTs, etc) are required. The first time of presenting an abbreviation begin with a full name or word (e.g., ante natal care) followed by corresponding abbreviation of such a word within the parenthesis (e.g., ANC).**

*We have reviewed the protocol and updated the abbreviations with full name*

**Tables 3, 4, 5, 6, 7 and 8 and Figure 3 should be moved into the supplement files so as to make the paper not too long.**

*We have uploaded the supplementary files during resubmission*

**Box 1: an abbreviation of Ministry of Health: MoH and MOH are not consistent. This should be either MoH or MOH through out**.

*We thank the Editor for this comment. It should be MoH and we have corrected it in the protocol.*

**If you would like to make changes to your financial disclosure, please include your updated statement in your cover letter. Guidelines for resubmitting your figure files are available below the reviewer comments at the end of this letter.**

*We would like to keep our financial disclosure at it’s current state.*

JOURNAL REQUIREMENTS:

**Please review your reference list to ensure that it is complete and correct. If you have cited papers that have been retracted, please include the rationale for doing so in the manuscript text, or remove these references and replace them with relevant current references. Any changes to the reference list should be mentioned in the rebuttal letter that accompanies your revised manuscript. If you need to cite a retracted article, indicate the article’s retracted status in the References list and also include a citation and full reference for the retraction notice.**

*We have checked our references and there is no need to replace any of them*
